# Acute infection as cause of hospitalization of asylum-seeking children and adolescents in Stockholm, Sweden 2015–2016

**DOI:** 10.1007/s00431-020-03795-1

**Published:** 2020-09-25

**Authors:** Olof Hertting, Joachim Luthander, Christian G. Giske, Rutger Bennet, Margareta Eriksson

**Affiliations:** 1 Pediatric Infectious Diseases Unit, Department of Pediatrics, Astrid Lindgren Children’s Hospital, Karolinska University Hospital, Stockholm, Sweden; 2 Department of Women’s and Children’s Health, Karolinska Institutet, Stockholm, Sweden; 3 Department of Clinical Microbiology, Karolinska University Hospital, Stockholm, Sweden; 4 Division of Clinical microbiology, Department of Laboratory medicine, Karolinska Institutet, Stockholm, Sweden

**Keywords:** Asylum seekers, Children, Adolescents, Infectious diseases, Hospitalization

## Abstract

We aimed to identify hospitalizations due to infectious diseases among asylum seekers and compare them to those of the resident population 1.6.2015–31.10.2016. Administrative numbers assigned to hospitalized non-resident children made them identifiable in the discharge register. The examined populations, expressed as person-years, were 334,573 residents and 7565 asylum seekers. There were 2500 episodes of infectious disease in 2240 resident children and 139 episodes in 121 asylum seekers. Among prevalent infections contracted before or during migration, there were 33 cases of tuberculosis, four of malaria, and one of louse-borne relapsing fever, all of which occurred in 13–17-year-old unaccompanied minors. Among younger asylum seekers, there were no significant differences in the spectrum of infectious discharge diagnoses compared to residents, but across all incident infections, 0–6-year-old asylum seekers had 3.2-fold and 7–12-year-old a 4.7-fold greater risk of being admitted. Screening for multidrug-resistant bacteria showed that 45/160 (28%) of the asylum seekers were colonized, but clinical infections caused by these species were rare.

*Conclusion*: There was a high rate of hospitalizations for acute infectious diseases in asylum-seeking children, but the spectrum and severity of infections were similar to that in resident children.

**Table:** 

**What is known:** *• Mental and physical health problems are common in immigrant children and adolescents.* **What is new:** *• Hospitalizations due to acute infections in asylum-seeking children and adolescents are common. In the context of this study, the severity and spectrum of infectious diseases seemed to be the same in the two groups; the increased hospitalization rate in asylum seekers may be due to social factors and perceived need for more support.*

**Electronic supplementary material:**

The online version of this article (10.1007/s00431-020-03795-1) contains supplementary material, which is available to authorized users.

## Introduction

The health status of refugee children has been a concern for public health, with special emphasis on chronic infections such as tuberculosis, hepatitis B, and HIV [1–4]. The risk for spread of antimicrobial resistance has also been discussed [5, 6]. Infections are common and are an increased burden to health care, in part due to the need for isolation precautions [7, 8].

In 2015, there was an unprecedented influx of refugees to Europe. Sweden, with a population of 9.9 million, received 162,877 asylum seekers (AS) that year, of which 70,384 were < 18 years including 35,369 unaccompanied minors (UAM). Of these children, 4656 were allocated to northern Stockholm. The mean time from arrival until the asylum process was completed increased from 142 to 320 days from 2014 to 2016 [9].

In Sweden, a national identity number is assigned to all citizens and individuals with a permanent or temporary residence permit, henceforward referred to as “residents.” Asylum seekers receive a temporary identity number when applying. However, in Stockholm Region, a personal administrative number is assigned to individuals without a national identity number such as AS and visitors when they are in contact with the health care system, making them identifiable in hospital registers. An administrative number is also assigned at hospitalization of children born in Sweden to AS parents, since these children are formally AS. Providers are reimbursed from the migration agency for health care costs of AS, making their identification mandatory. All health care of children, including AS, is provided without cost to patients.

National and international recommendations were issued for the recognition, treatment, and screening of newly arrived refugees for chronic infections [10]. EU recommendations were recently summarized [11].

Health assessment of AS in Stockholm is performed at specially designated outpatient screening centers. We previously published a detailed report of tuberculosis infection and disease among UAM seeking asylum in northern Stockholm in 2015 [4]. In hospitalized children, screening for infections is performed only when clinically indicated. At this stage of their asylum process, immunization status is not available. After being granted asylum and permanently allocated to a residential area, they are included in the immunization program.

In this study, we investigated pediatric hospitalizations for infectious diseases for 16 months 2015–2016, to compare AS children and adolescents with the resident population.

## Methods

In Stockholm Region in 2015, 8.8% (42,537/485,687) of the resident population < 18 years were foreign-born, out of which 41% (17,271/42,537) were born in in Asia, most commonly Iraq and Syria, and 14% (5834/42,537) in Africa, most commonly from the Horn of Africa region. Immigrants reuniting with resident families receive personal identity numbers within weeks after arrival, making them a part of the resident population.

Astrid Lindgren Children’s Hospital is a tertiary referral center with a primary catchment area comprising northern Stockholm with a population of 250,930 children < 18 years in 2015. From the hospital databases, we retrospectively retrieved information about hospitalizations from 1 July 2015 to 31 Oct 2016. We identified all children with a discharge diagnosis of infection. Tuberculosis, malaria, and relapsing fever in AS were considered prevalent, i.e., present at arrival; all other clinical infections were considered incident. Hospitalizations in neonatal and oncology wards were excluded because of the specific nature of infections in these patient groups. We reviewed charts of admitted children with administrative numbers to exclude visitors and to verify the discharge diagnosis. The charts, including microbiological reports, were comprehensively reviewed by one of the authors, who are all senior pediatricians. As a measure of severity, we recorded the number of episodes leading to intensive care admission. We recorded colonization with extended-spectrum beta-lactamase (ESBL)–producing enteric bacilli and methicillin-resistant *Staphylococcus aureus* (MRSA) among all AS.

Countries of origin were grouped according to the UN geographical subregions [12].

To estimate the AS population < 18 years in our area, we used monthly rates of asylum applications in persons crossing the border and assigned to Stockholm Region from January 2015 through October 2016 as published on the website of the Swedish Migration Agency [9]. The available age strata were 0–6, 7–12, and 13–17 years, which we used for the disaggregated analysis of discharge diagnoses. To obtain a population denominator for AS children, we used the mean time from arrival until an asylum decision was reached to calculate the number of person days within the study period (Online Resource 1). We restricted the estimation of rates of incident infections to children < 13 years since the social background and living conditions of the UAM teenagers differed fundamentally from those of the younger children. For resident children, we used population data from Statistics Sweden at December 31, 2015, multiplied by 16/12 to obtain the number of person-years during the 16-month observation time [13]. Children born to AS parents after arrival in Sweden were omitted from the incidence calculations since they were not included in the population denominator.

We used chi-square tests when comparing proportions and the online facility at www.medcalc.org for calculating risk ratios. The study was approved by the Stockholm regional ethics committee (reference number 2017/1259).

## Results

**Fig. 1:**
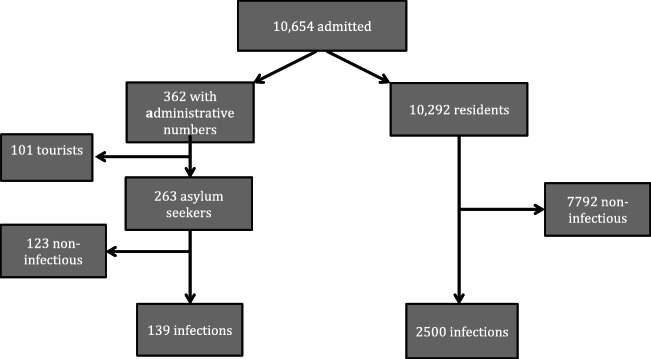
Study flow chart

The flow chart (Fig. 1) shows the number of children at each step of the inclusion process.

A discharge diagnosis of an infection was found in 2500/10,292 episodes (24%) in residents and 139/263 episodes (53%) in AS (*p* < 0.001). They occurred in 2240 resident and 121 AS children, respectively, resulting in 1.12 and 1.13 episodes per child (difference not significant).

The regions of origin of AS children in the three age groups are displayed in Table 1. In the 0–6-year group, 22/59 (39%) of the children were born in Sweden. Of these, 16 were infants, three were one, two were two, and one was three years old. Most common nationality among AS children < 13 years was Syrian (14/68, 21%) and of children ≥ 13 years Afghan (29/51, 57%). All children in the 13–17 age group were UAM. The number of UAM among the younger children, all in foster care, was unknown.

**Table 1:** Regions of origin of 121 asylum-seeking children hospitalized with infection at least once

Age group	0–6		7–12	13–17
UN subregion	Foreign-born	Swedish-born		
E. Europe	1	1		
S. Europe	2			
N. Africa	1		1	
W. Africa		1		
E. Africa	3	6	2	17
M. Africa	1			
W. Asia	14	3	5	2
C. Asia	1	4	1	
S. Asia	7	3	2	30
E. Asia	1	4	1	
SE. Asia	4			
Caribbean	1			
Unknown	1			1

The age and sex distribution are presented in Table, Online Resource 2. Among resident and AS children, < 13 years the age and sex distribution were similar, but in the disproportionally large group of AS teenagers, all of whom were UAM, 43/50 (86%) were male.

Numbers and admission rates for infectious diseases by age group are presented in Table 2. The admission rates for asylum seekers were significantly higher in AS than in residents.

**Table 2:** Comparison of episodes of hospitalization for infection in residents and foreign-born asylum seekers. Hospitalization rate is calculated per 1000 person-years

Age	Residents			Asylum seekers			Rate ratio
	Pop.	*n*	Rate	Pop.	*n*	Rate	(95% C.I.)
0–6	140,705	1990	14	1044	48	4.6	3.2 (2.4–4.2)
7–12	112,727	334	3.0	704	12	17	4.7 (3.2–10.1)
13–17	81,141	176	2.2	5817^a^	56^a^*	10	4.4 (3.3–5.9)

*Pop.*, population (person-years); *C.I.,* confidence interval. All rate ratios are significant (*p* < 0.001)

^a^Entire Stockholm Region

In Table 3, some discharge diagnoses among residents and AS are compared. The spectrum of incident infections did not significantly differ between the two groups. Of prevalent infections, 33 AS children had tuberculosis, five had malaria, one had *Echinococcus*, and one had louse-borne relapsing fever (*Borrelia recurrentis*). All children with bacterial gastroenteritis were residents.

**Table 3:** Numbers, rates, and relative risk of hospitalization for some discharge diagnoses

	Residents Pop. 253,432	Asylum seekers Pop. 1748	Asylum seekers born in Sweden	Relative risk 95% C.I.
Gastroenteritis (A00–09)	226 (0.9)^a^	11 (6.3)^a^	2	7.0 (3.8–13)***
Varicella (B01)	22 (0.1)	3 (1.7)	2	20 (5.9–66)***
Viral infection, unspecified (B34.9)	154 (0.6)	3 (1.7)	1	2.9 (0.9–9.0)
Upper respiratory infection (J06)	136 (0.5)	6 (3.4)	3	6.4 (2.8–14)***
Influenza (verified, J10)	67 (0.3)	4 (2.3)	1	8.6 (3.2–24)***
All-cause pneumonia (J12–J18)	324 (1.3)	8 (4.6)	2	3.6 (1.8–7.2)***
Empyema (J86)	2 (0.01)	2 (1.1)	2	145 (20–1028)***
Skin abscess (L02–L03)	87 (0.3)	3 (1.7)	1	5.0 (1.6–16)**
Urinary tract infection (N10, N39)	138 (0.5)	1 (0.6)	4	1.1 (0.1–7.5)
Tuberculosis (A15–A17)	3	33^b^		
Malaria	0	5^b^		

Populations (Pop.) are person-years in children < 13 years. The population of asylum seekers born in Sweden was unknown. **P < 0.01; ***P < 0.001

^a^Incidences rates (cases/1000 person-years)

^b^Includes asylum-seeking children <17 years from entire Stockholm County

A total of 101 children with a diagnosis of infection were admitted to intensive care, 98 resident, and three AS. The main reason was respiratory infection (66/101, 65%). Of the three AS patients, there were one infant with respiratory syncytial virus infection and two children with chronic comorbidity and respiratory infections.

Of all AS children, 61% (160/263) were screened for resistant bacteria on admission; 27/160 (17%) were colonized with ESBL-producing enteric bacilli and 19/160 (12%) with MRSA.

Clinical isolates of ESBL-producing *Escherichia coli* were found in 36 children, one among the AS (culture taken at appendectomy) and 35 among the resident children (11 urine, 19 appendectomies, and 3 anal abscesses). Clinical isolates with MRSA were found in three AS (two skin abscesses and one orbital abscess) and 9 resident children (all skin abscesses).

## Discussion

In this study of AS children arriving in Sweden 2015–2016, we have shown that those allocated to our area were more often hospitalized because of infections compared with resident children. This is in line with an earlier study from Sweden where an excess risk for all-cause hospitalizations in immigrant children < 5 years was reported [14].

We could find four recent retrospective pediatric studies of diagnoses among refugees, two from Europe and two from Turkey [15–18]. As in our study, infectious disease diagnoses dominated. In two European studies of young adults, infectious disease was an important reason for hospitalization and in one of them a relative increase from 33% in 2004 to 56% in 2014 was observed [7].

Respiratory infections, gastroenteritis, and skin infections were the most common diagnoses in both AS and residents. It is probable that social factors such as language barriers and uncertainty about the availability and quality of supportive care in the home lowered the threshold for admission of AS with common infections. A similar observation was shown in a German register–based study, suggesting that AS were more likely to be admitted for conditions that could have been managed in primary care, so-called avoidable hospitalizations [19]. The same was found among children 1–4 years of age born to mothers from high migratory pressure countries compared to children born to Italian mothers [20].

Using admission to PICU as a proxy measure of severe disease, there was no difference between resident and AS children.

Older children in the UAM category were most often hospitalized to diagnose and initiate treatment of tuberculosis. In this group, we also found other infections contracted during migration: four with malaria and one with louse-borne relapsing fever. In screening studies of recently arrived UAM migrants to Germany, a high prevalence of intestinal parasites was reported [21, 22]. We did not routinely study this in our hospital.

In the AS group, there were more cases of varicella. We interpreted this to be a consequence of a relatively large fraction of older children from Asia and Africa being susceptible to varicella and the crowded living condition in refugee shelters. We did not record any outbreaks as reported from Germany, where the cost effectiveness of immunization programs was also discussed [23].

We do not know if the higher rate of complicated pneumonia such as empyema was just a coincidence or could be due to delayed health-seeking behavior by parents to asylum-seeking children.

Most studies on antibiotic resistance among refugees report colonization and not isolates from clinical infections (6). Almost one-third of the screened AS in this study were carriers of ESBL-producing bacteria or MRSA. We have no information about our pediatric resident population, but 101/2134 (4.7%) of a mixed Swedish population were shown to carry ESBL-producing *Escherichia coli* [24]*.* One-third of 175 Swedish tourists returning from various continents carried ESBL-producing bacteria at least transiently [25].

One AS had an isolate of ESBL-producing bacteria found at appendectomy. During the same period, 35 resident children had clinical isolates of ESBL-producing enterobacteria, 19 of which were from appendectomies, 11 from urine, and 3 from anal abscesses. The incidence of clinical infections due to drug-resistant bacteria is in accordance with a study from a German hospital [17]. For MRSA, the situation was more complex with clinical infections being frequent (3/12) among AS.

A strength of our study is our attempt to correlate our results with a population base of both residents and AS. The calculation of AS children population is not easy. The time from arrival in Sweden until an asylum decision is reached is highly variable and may extend to several years, as illustrated by the age of Swedish-born AS. Using means compensates for this variability.

Another limitation in our study is that we reviewed the hospital charts only in hospitalized children. Many important health problems are managed in outpatient clinics, where also screening for hepatitis B, HIV and tuberculosis is offered.

It is important to follow this vulnerable group of children and adolescents where we know that both chronic diseases and mental health problems are more prevalent and may present later [26]. Many also continue to stay in crowded living conditions that could potentially increase the risk of contracting and spreading infectious diseases. Our results demonstrate a high rate of hospitalizations for acute infectious diseases in AS children, but the spectrum and severity of infections were similar to that of resident children.

## Electronic supplementary material

ESM 1 (DOCX 16 kb)

ESM 2 (DOCX 14 kb)

## Abbreviations

ESBL: Extended-spectrum beta-lactamase
HIV: Human immunodeficiency virus
ID: Personal identity number
MRSA: Methicillin-resistant *Staphylococcus aureus*
TB: Tuberculosis
T-ID: Temporary personal identity number
UAM: Unaccompanied minors
