# Supplementary material for: Acute infection as cause of hospitalization of asylum-seeking children and adolescents in Stockholm, Sweden 2015–2016
Source: Eur J Pediatr. 2020 Sep 25;180(3):893–8. doi: 10.1007/s00431-020-03795-1 (PMC7886722; doi:10.1007/s00431-020-03795-1)
Supplement: Supplementary file 1 — (DOCX 16 kb) [file 431_2020_3795_MOESM1_ESM.docx]

# **Acute infection as cause of hospitalization of asylum-seeking children and adolescents in Stockholm, Sweden 2015-2016, European Journal of Pediatrics**

^*^Olof Hertting, Joachim Luthander, Christian G. Giske, Rutger Bennet, Margareta Eriksson,

## *Corresponding author: Olof Hertting, olof.hertting@sll.se, Ph. +46 73 941 53 27

Online Resource 1. Calculation of population denominator within the study period July 2015—October 2016.

1. Asylum seeking children arriving in Sweden

|  | 2015 (220 days*) | | 2016 (320 days*) | |
| --- | --- | --- | --- | --- |
|  | N | Person days** | N | Person days** |
| Jan | 1 483 | 59320 | 1798 | 539400 |
| Feb | 1 328 | 92960 | 1063 | 287010 |
| Mar | 1 294 | 129400 | 784 | 188160 |
| Apr | 1 162 | 151060 | 700 | 147000 |
| May | 1 950 | 312000 | 691 | 124380 |
| Jun | 2 552 | 484880 | 733 | 109950 |
| Jul | 3 210 | 706200 | 775 | 93000 |
| Aug | 5 134 | 1129480 | 877 | 78930 |
| Sep | 9 740 | 2142800 | 957 | 57420 |
| Oct | 17 495 | 3848900 | 941 | 18820 |
| Nov | 18 155 | 3994100 |  |  |
| Dec | 6 881 | 1513820 |  |  |

*Mean asylum application processing time to decision
** Processing time falling within the study period

The processing time was allocated to the age strata 0—6, 7—12 and 13—17 years in the same proportions as the numbers of applying children. The Stockholm fraction was considered equal to the fractions of children in each age group initially allocated to Stockholm Region in 2015 and 2016, respectively. Of these, 50% were considered resident in northern Stockholm, which has half of the population in Stockholm Region.

Transfers in and out of Stockholm were assumed equal.

1. Resulting population denominators

| Age group | Person years |
| --- | --- |
| 0—6 | 1044 |
| 7—12 | 704 |
| 13—17 | 2908 |
