# Supplementary material for: Acute infection as cause of hospitalization of asylum-seeking children and adolescents in Stockholm, Sweden 2015–2016
Source: Eur J Pediatr. 2020 Sep 25;180(3):893–8. doi: 10.1007/s00431-020-03795-1 (PMC7886722; doi:10.1007/s00431-020-03795-1)
Supplement: Supplementary file 2 — (DOCX 14 kb) [file 431_2020_3795_MOESM2_ESM.docx]

# **Acute infection as cause of hospitalization of asylum-seeking children and adolescents in Stockholm, Sweden 2015-2016, European Journal of Pediatrics**

^*^Olof Hertting, Joachim Luthander, Christian G. Giske, Rutger Bennet, Margareta Eriksson,

## *Corresponding author: Olof Hertting, olof.hertting@sll.se, Ph. +46 73 941 53 27

Online Resource 2. Age and sex of residents and asylum seekers hospitalized with infection.

| Age | Residents | | Asylum seekers | |
| --- | --- | --- | --- | --- |
| (years) | M | F | M | F |
| 0 | 406 | 279 | 12 | 14 |
| 1 | 216 | 188 | 7 | 7 |
| 2 | 128 | 99 | 8 | 2 |
| 3 | 77 | 72 | 1 | 2 |
| 4 | 69 | 61 |  | 3 |
| 5 | 56 | 37 |  | 1 |
| 6 | 52 | 36 | 2 |  |
| 7 | 48 | 31 | 2 | 1 |
| 8 | 24 | 31 | 1 |  |
| 9 | 23 | 19 | 1 |  |
| 10 | 25 | 22 | 1 |  |
| 11 | 21 | 16 | 2 | 2 |
| 12 | 23 | 19 | 2 |  |
| 13 | 20 | 22 | 1 |  |
| 14 | 24 | 19 | 8 |  |
| 15 | 15 | 17 | 12 | 2 |
| 16 | 10 | 15 | 18 | 2 |
| 17 | 8 | 12 | 4 | 3 |
